# 1790. Characteristics of Patients with Mpox Treated with Tecovirimat during the 2022 Outbreak in Puerto Rico

**DOI:** 10.1093/ofid/ofad500.1619

**Published:** 2023-11-27

**Authors:** Hector J Melendez Gonzalez, Melissa Marzan Rodriguez, Iris Cardona Gerena

**Affiliations:** Puerto Rico Department of Health, San Juan, Puerto Rico; Puerto Rico Department of Health, San Juan, Puerto Rico; Puerto Rico Department of Health, San Juan, Puerto Rico

## Abstract

**Background:**

In June 2022, the first case of Mpox, was diagnosed in Puerto Rico, followed by a sharp increase in cases that peaked in August 2022. In total, 108 cases were confirmed during this period. Throughout this time period, Tecovirimat (Tpoxx) was already available upon request to the Puerto Rico Department of Health (PRDoH). In this report we aim to describe the clinical characteristics and outcomes of all the patients in Puerto Rico who received Tecovirimat.

**Methods:**

A cross sectional study was conducted to describe clinical and epidemiological profile of Mpox patients who received Tecovirimat. We use the data collected from our institution (PRDoH), who was responsible for the approval, delivery and monitoring of Tecovirimat prescription during the outbreak. The analysis period ranges from August 2022 to November 2022. Univariate analysis was conducted, including the median and percentage, to provide a summary of the distribution of the variables of interest.

**Results:**

We present the clinical characteristics of 27 male patients who received Tecovirimat for a diagnosis of Mpox during the outbreak in Puerto Rico. Most patients were treated during August and September (85%), followed by October and November 2022 (15%). Most received treatment in outpatient settings at private clinics (63%) followed by government clinics (37%). Of the patients that required Tecovirimat, 67% had human immunodeficiency virus (HIV) and the median age was 32 years. Prior immunization with smallpox vaccine was reported by two patients, while 70% were not immunized and the immunization status of 22% was unknown. The most common indications for Tecovirimat prescription were genital structure involvement (67%), severe pain (37%), oral cavity involvement (22%), proctitis (19%), and immunosuppression (11%). Only two patients received Tecovirimat while admitted to an acute care setting. No severe adverse events or deaths were reported among these patients, and there were no suspected cases of Tecovirimat resistance.

**Conclusion:**

This study provides relevant clinical information on the use of Tecovirimat in patients with Mpox during the outbreak in Puerto Rico. These findings may have important implications for the clinical management of future outbreaks of Mpox and other related infections.

**Disclosures:**

**Iris Cardona Gerena, MD**, Valneva: Honoraria

